# Development and results of the epilepsy surgery in Armenia: hope for a better future

**DOI:** 10.3389/fneur.2023.1152275

**Published:** 2023-08-21

**Authors:** Biayna Sukhudyan, Krasimir Minkin, Sevak Badalyan, Kaloyan Gabrovski, Ani Gevorgyan, Irina Tovmasyan, Ara Babloyan, Petia Dimova

**Affiliations:** ^1^Arabkir Joint Medical Center, Yerevan, Armenia; ^2^University Hospital St. Ivan Rilski, Sofia, Bulgaria

**Keywords:** epilepsy surgery, drug-resistant epilepsy, lesional epilepsy, development of epilepsy surgery, epilepsy in developing countries

## Abstract

**Purpose:**

We present our experience with the national epilepsy surgery program in Armenia by tracing the development of epilepsy surgery in the largest pediatric neurology department at “Arabkir” Medical Center. This development was possible on the basis of a strong collaboration with the Epilepsy Surgery center at the University Hospital “Sofia St. Ivan Rilski,” Sofia, Bulgaria.

**Materials and methods:**

Our material included 28 consecutive patients with lesional drug-resistant epilepsy evaluated. All patients underwent 3 T MRI and Video-EEG monitoring. Brain 18FDG-PET was done in 13 patients in St. Petersburg. Fifteen patients (53%) had preoperative neuropsychological examination before surgery. All operations were done by the same neurosurgical team on site in Arabkir Hospital.

**Results:**

The majority of the patients in our cohort benefited from the epilepsy surgery as 25 (89%) are free of disabling seizures (Engel class I) and three patients (11%) did not improve substantially (Engel class IV). Eleven patients (39%) are already ASM-free after surgery, 4 (14%) are on monotherapy, 11(39%) get two drugs, and 2(7%) are on polytherapy, one of them still continues having seizures. In 12 patients (43%), we were able either to withdraw therapy or to decrease one of the ASM.

**Conclusion:**

We believe that, although small, yet encompassing patients along the usual age spectrum and with the most frequent pathologies of drug-resistant epilepsies, our experience can serve as a model to develop epilepsy surgery in countries with limited resources.

## Introduction

Armenia is a developing country located in Southern Caucasus with a population of 3 million people. In early 2018, the World Bank upgraded Armenia’s status from a “lower middle-income” to an “upper middle-income” nation, with health expenditure *per capita* reaching 524 USD.^1^ Given the epilepsy prevalence rate of 5 per 1,000 person-years (1), the estimated number of people with active epilepsy in the country is 15,000 at any given point. Considering that about 30% of these patients have drug-resistant epilepsy and half of them are potential candidates for epilepsy surgery (2), there are at least 2,000 surgical candidates. Currently, there are over 200 adult neurologists, 40 pediatric neurologists, and 40 neurosurgeons working in the country, and two epilepsy centers serve the pediatric and adult population.

Epilepsy surgery has proven to be very effective in treating refractory focal epilepsies in children and adults by achieving seizure freedom or seizure control well beyond any other medical or dietary therapies (3). It still remains inaccessible in countries with limited resources for economic reasons and lack of well-organized epilepsy surgery centers. A 2006 survey conducted by the World Health Organization, the International League Against Epilepsy, and the International Bureau of Epilepsy found that epilepsy surgery was available in only 13% of Low Middle Income Countries (LMIC) as compared to 66% of high-income countries (4).

Here, we present our experience with the national epilepsy surgery program in Armenia by tracing the development of epilepsy surgery in the largest pediatric neurology department at “Arabkir” Medical Center and by analyzing our results in the cohort of operated patients for the period 2016–2022. This development was possible on the basis of a strong collaboration with the Epilepsy Surgery center at the University Hospital “Sofia St. Ivan Rilski,” Sofia, Bulgaria.

## Materials and methods

Our material included 28 consecutive patients with drug-resistant epilepsy evaluated and operated on in Arabkir Medical Center, Armenia between 2016 and 2022. Drug-resistant epilepsy was defined as the failure of two tolerated, appropriately chosen and used antiepileptic drugs (AEDs) to achieve sustained seizure freedom (5). All patients had a lesion on the brain MRI and underwent long-term video-EEG monitoring (LT-VEEG) in our Epilepsy Monitoring Unit. The LT-VEEG was performed for minimum 24 to maximum 48 h, with anti-seizure medication (ASM) withdrawal or transient ASM reduction before and during the examination. Brain ^18^FDG-Positron Emission Tomography (PET) was done in 13 patients in St. Petersburg (“Institute of Human Brain”).

Fifteen patients (53%) had preoperative neuropsychological examination before surgery by Epitrack or EpiTrack Junior, Boston Naming Test (BNT), Grober & Buschke verbal memory test, Beck Depression Inventory, Rey Complex Figure Test, Montreal Cognitive (MoCa) Test, Children’s Memory Scale (CMS), and Wechsler Intelligence Scale for Children (WISC V) (Table 1). The results showed full concordance with EEG and MRI data. Due to lacking financial coverage neuropsychological testing, including post-operative assessment was not possible in all patients.

**Table 1:** The results of presurgical neuropsychological assessment.

Pathology	Side	Intelligence (*N* = 4)	Visuo-spatial memory (*N* = 14)	Language (*N* = 13)	Executive function (*N* = 14)	Verbal memory (*N* = 15)	Mood (*N* = 1)
HS	R		3	2	3	2	
	L		3	2	5	3	1
Residual change	R						
	L	2		2	2	2	
FCD	R				2	1	
	L	1			1		
LEAT	R						
	L	1		2	1		

The results from the presurgical work-up were discussed in multidisciplinary on-line conferences including neurologists, neurosurgeons, and neuropsychologists from both centers. The type and extent of surgical resection were defined according to the limits of the presumable epileptogenic zone (EZ) and its relationships with the functional zones. If invasive EEG exploration was indicated for precise localization of EZ, its relationships with eloquent cortex, and the feasibility of a tailored surgical resection (6) the patient was referred for SEEG and surgery to Epilepsy Surgery Center in Sofia.

All operations were done by the same neurosurgical team (KM, KG, and SB) on site, in Arabkir Hospital. All operated patients underwent LT-VEEG and control MRI 2 months after surgery and had a regular follow-up visits every 6 months thereafter.

Surgery outcome was assessed by Engel Outcome Scale (7) and classified into following categories: Engel class I (free of disabling seizures), Engel class II (rare disabling seizures [“almost seizure-free”]), Engel class III (worthwhile improvement), and Engel class IV (no worthwhile improvement). As “disabling” we qualified focal seizures with impairment of consciousness and focal to bilateral tonic–clonic seizures.

## Results

A total of 28 epilepsy surgeries (16 pediatric cases) were done between September 2016 and March 2022. The mean follow-up period was 4 years (1 year–7 years). The patients (14:14 sex ratio) were at an age from 2 to 37 years (mean 17 years). Epilepsy onset was in the age range 1–19 years (mean 6 years), with six of the patients (21%) experiencing seizures in the first year of life. The mean duration of epilepsy was 10 years (range 1–33 years). Ten patients (36%) had daily, 8 (29%)—weekly, and 10 (36%)—monthly seizures. Seizures during LT-VEEG were recorded in 25 patients (89%). In three patients with tumors and rare seizures, we considered well-localized and corresponding to the lesion (lateralization and localization) interictal discharges on LTM-VEEG sufficient to propose surgical intervention.

Seven patients (25%) were preoperatively receiving one ASM, 15 patients (54%)—two ASM, and 6 (21%) were on polytherapy with ≥3 ASM. Co-morbidities were present in 10 cases (35%): four patients (14%) had intellectual disability, five patients (18%) had learning disability, and one child (4%) had autism.

The baseline characteristics of patients are presented in Table 2.

**Table 2:** Baseline characteristics of patients.

	Total number of patients—28
Febrile seizures	10 (35%)
Co-morbidities	10 (35%)
Types of seizures	
Focal seizures	12 (43%)
Focal and focal-to-bilateral	9 (32%)
Focal-to-bilateral only	3 (11%)
Spasms	4 (14%)
Investigations	
Ictal EEG	25 (89%)
3T MRI	27 (96%)
1.5 T MRI	1 (4%)
PET	13 (46%)
Genetics	1 (4%)
Pathology	
LEAT	7 (25%)
*DNET*	3 (43%)
*Pilocytic astrocytoma*	1 (14%)
*Isomorphic astrocytoma*	1 (14%)
*Pilomyxoid astrocytoma*	1 (14%)
*Ganglioglioma*	1 (14%)
FCD	8 (28%)
*Temporal*	4 (50%)
*Frontal*	3 (37%)
*Hemispheric*	1 (13%)
HS	10 (36%)
Residual posthypoxic-ischemic/posthemorrhagic	3 (11%)
Type of surgery	
Tumor resection	7 (25%)
ATLE + AHE	10 (39%)
*Hemispherotomy*	2 (7%)
*Tailored disconnection*	2 (7%)
Lesionectomy	7 (25%)
Surgeries	
Temporal	19 (68%)
Extratemporal	5 (18%)
Temporal + Extratemporal	4 (14%)
Outcome	
I	25 (89%)
II	-
III	-
IV	3 (11%)
Free of ASM	11 (39%)

All patients had focal lesional epilepsy, including four children (14%) with hemispheric pathology. Nineteen patients (68%) had temporal lobe epilepsy (nine left-sided and 10 right-sided lesions), and five patients (18%) had extratemporal epilepsies. The most frequent histopathological finding was hippocampal sclerosis (10 cases), followed by focal cortical dysplasia (FCD; eight cases), Long-term Epilepsy Associated Tumors (LEATs; seven cases), and residual post-hypoxic–ischemic/post-hemorrhagic changes (three cases). Epilepsy surgery was temporal in 19 (68%) and extratemporal in 5 (18%). In four cases (14%), we performed disconnective surgeries: hemispherotomy (*n* = 2) and tailored disconnections (*n* = 2).

In two patients, we performed intraoperative neuromonitoring (IONM) under general anesthesia for mapping of motor cortex and corticospinal tract, and in one patient IONM was done in awake condition for language mapping.

**Table 3:** Complex epilepsy surgery cases with IONM.

	Clinical data	Pre-operative MRI	Post-operative CT/MRI
Patient 1	Pathology: Residual left-hemispheric pathology.	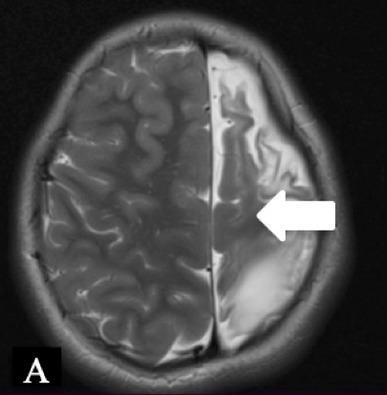	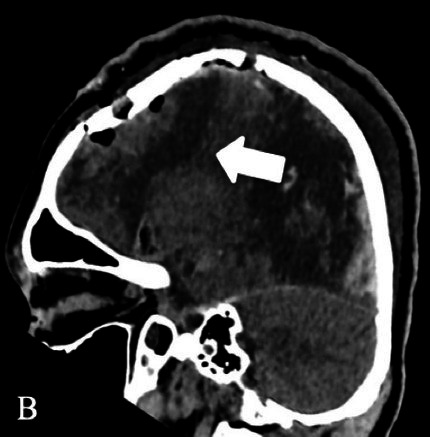
M, 12 years	Seizures: Daily asymmetric tonic spasms provoked by unexpected tactile and auditory stimuli.		
	Neurological examination: Mild right-sided hemiparesis.		
	MRI: Axial T2 with a small cortical remnant on the left parasagittal (white arrow in Figure A).		
	Wada test: Confirmed right leg motor functionality of the parasaggital cortical remnant.		
	Surgery: Modified hemispherotomy after intra-operative monitoring (IONM) and preservation of the right leg motor function (early sagittal CT showing the descending fibers from that area, white arrow on Figure B).		
	Follow-up: Early postoperative seizures for 1 week, then >1 year seizure-free.		
Patient 2	Pathology: Large cortical malformation of the left hemisphere (white oval on axial T2-MRI, Figure A1).	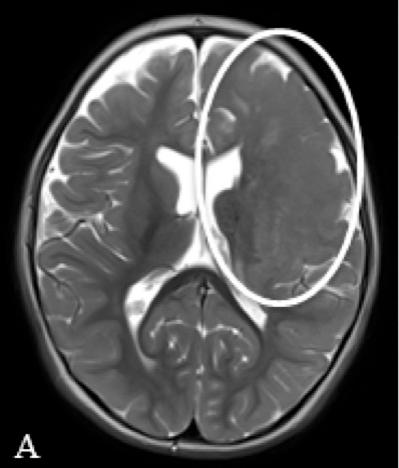	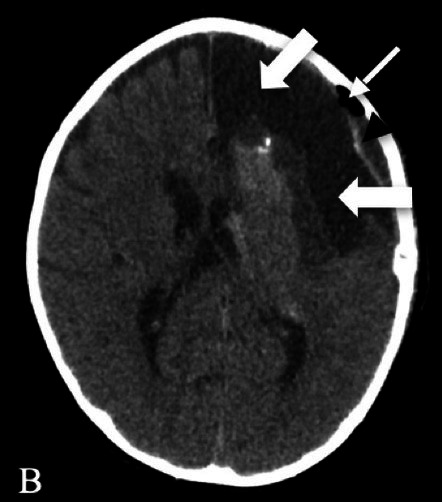
F, 2 years	Seizures: Daily asymmetric spasms and right hemifacial seizures		
	Neurological examination: Normal		
	Surgery: IONM during extensive frontal lobectomy up to the motor cortex; and temporal disconnection. Early postoperative axial CT scan (Figure B1) demonstrating the cystic cavity on the left frontal (bold white arrow) and small subdural hygroma with air collection (thin white arrows).		
	Follow-u p: No motor deficit. Early postoperative seizures with excellent effect of Carbamazepine introduction.		
Patient 3	Pathology: Left parietal tumor adjacent to eloquent areas (supramarginal gyrus) as seen on sagittal T2-MRI (hyperintensity lesion, white arrow, Figure A2).	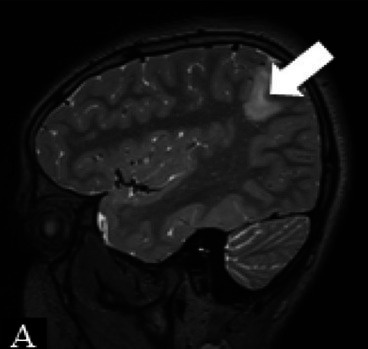	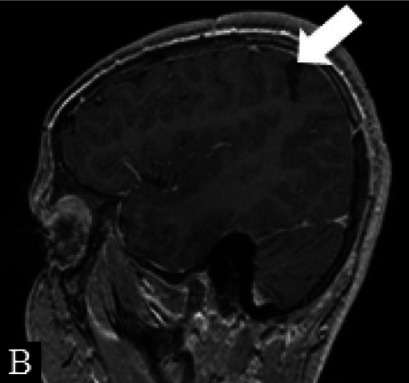
	Seizures: Head deviation to the right with bilateralization.		
M, 13 years	Surgery: Awake craniotomy with IONM and simultaneous testing of language functions allowed complete tumor resection (white arrow on sagittal and thin cystic cavity on 6-month post-operative contrast-enhanced T1-MRI, Figure B2).		
	Follow-up: Seizure-free and no ASM for >5 years.		

### Epilepsy surgery outcome

Majority of our patients benefited from the epilepsy surgery as 25 (89%) were free of disabling seizures (Engel class I) and three patients (11%) did not improve substantially (Engel class IV). Among the seizure-free patients, 17 (68%) had temporal lobe epilepsy, 3 (12%)—frontal lobe epilepsy, 2 (8%)—parietal lobe epilepsy, and 3 (12%)—hemispheric epilepsy.

The best outcome was observed in the “residual lesions” group, where all three patients have Engel class I outcome, followed by HS group (nine out of 10 seizure-free), FCD group (seven out of 8 seizure-free), and LEATs (six out of seven seizure-free).

In our patients with excellent post-surgical outcome an epilepsy duration of less than 10 years was almost twice as prevalent as a longer epileptic disorder (17 vs.11 patients).

We were able to stop ASM in 11 patients (39%), while four patients (14%) are on monotherapy, 11 (39%)—on two drugs, and two (7%)—on polytherapy (one still having seizures).

Five patients (18%) experienced early post-operative seizures during the first week after operation. In none of them treatment was stopped. In one patient with big dysplasia and tailored disconnective procedure (“everything-but-motor”; case 2, Table 3), the change in ASM led to >1-year seizure control; therefore we could consider this case made “drug-sensitive” by the epilepsy surgery. In three patients the seizures spontaneously stopped.

We had three cases of epilepsy surgery failure: (1) one adult with right-sided HS, concordant results of all presurgical examinations, right ATLE, and presumable “temporal-plus” epilepsy due to probable insular involvement (not possible to invasively explore at the time of the epilepsy surgery); (2) one child with right frontal FCD, complete removal of the visible lesion and 1-year seizure-freedom without ASM, with a relapse of new seizure type suggesting a larger EZ; (3) one child with complete removal of a right temporal isomorphic astrocytoma, 1-year seizure freedom, relapse on withdrawal of ASM, whose re-introduction was not efficient, thus suggesting a genetic epilepsy background (not previously excluded as the patient had only negative *SCN1A* sequencing due to febrile seizure clusters preceding the epilepsy).

The results of surgeries are presented in Table 2.

### Post-surgical complications

None of the patients had persistent postoperative neurological deficit. In two cases, transient motor deficit due to SMA syndrome and/or edema resolved in 2–4 weeks.

We have observed unusual postoperative fever in two patients (7%). The elevated cerebro-spinal fluid (CSF) pleocytosis prompted antibacterial treatment for meningitis in these cases, although no bacterial microorganism was detected in the CSF culture. Another patient (4%) had local staphylococcal facial skin infection adjacent to a pin insertion site and was treated with antibiotics.

In two patients (7%) with large disconnective surgeries signs of increased intracranial pressure (somnolence, weakness, and bilateral abducens palsy) required a lumbar tap that proved a 2-fold increase in CSF pressure. In one of these patients the lumbar puncture led to a dramatic improvement, whereas the other needed a ventriculo-peritoneal shunting. No deaths or new neurological deficits were related to the epilepsy surgery.

## Discussion

### Development of epilepsy surgery program

The epilepsy surgery program in Armenia was not blindly modeled after patterns followed in the Western world, where the epilepsy surgery centers (e.g., in most European countries) developed in neurosurgery or neurology departments (8). To the best of our knowledge, Armenia stands as the first example of an epilepsy surgery program that emerged within a pediatric neurology department. This accomplishment was possible due to the prioritization of our hospital’s project, “Improving epilepsy patients’ care.” For more than 10 years, the Pediatric Epilepsy Center provides care for over 3,600 Armenian children with epilepsy.

Lack of resources and trained staff appeared a huge hindrance toward starting a specialized epilepsy surgery program (9–11). In our settings, the well-trained epilepsy specialists could define patients eligible for surgery, yet for many years the lack of LT-VEEG, high quality MRI, and of neurosurgery department contributed to the substantial delay in providing a thorough presurgical work-up and surgical treatment for drug-resistant patients (Table 3).

During this period, we made a remarkable progress in the presurgical evaluation work-up (phase 1). We started with a 32-channel video-EEG workstation and between 2017 and 2019 our center was equipped by four video-EEG stations, including one 64-channel. One local neurosurgeon and a neurosurgery trainee have joined our epilepsy team. The local epilepsy specialist, neurosurgeon, and EEG technician accomplished various training programs at epilepsy and neurosurgery centers in Europe and the United States, including multiple visits to our partner, the University Hospital “St. Ivan Rilski” in Sofia. Two experienced neurodevelopmental pediatricians received virtual and in-person training from experts in Bulgaria and the United States in neuropsychological testing, including testing during the Wada procedure. Furthermore, the newly established neurosurgery department at the “Arabkir” Medical Center has undergone significant and continuous upgrades.

### Indications and pathology

We have restricted our selection criteria to well-defined lesional cases. This approach reflected the stepwise experience of epilepsy surgery programs in the developed countries that started with resections of structural epileptogenic lesions and had high success rates due to good correlation of the lesion and the EZ (8, 12, 13). In the selected, lesional only, cases excellent postoperative outcome was highly probable even by relatively inexpensive and non-invasive technologies. As already emphasized, this approach primarily depends on locally available settings and expertise without compromising the patient safety (8).

In our cohort HS (63%; adults), FCD (23%; children), and LEATs (23%), were the most common pathologies, which is a finding fully concordant with the results of experienced centers (14). Although we aimed to obtain ictal EEG recordings in most patients, in three cases with LEATs surgeries were performed based on interictal EEG findings and led to seizure-freedom. Our confidence relied on already described good correlation of seizure-onset zone with structural abnormalities seen in MRI in tumor-related epilepsies (9, 15–17).

In high risk surgeries where EZ may possibly overlap with eloquent cortex, brain mapping with functional MRI and invasive evaluation by SEEG or subdural grids could help in preoperative planning of the resection. Inborn and acquired lesions may provoke functional reorganization and important changes in the map of eloquent cortex and in such cases awake surgery and/or IONM are important options to intraoperatively adapt the preoperative plan in order to avoid permanent postoperative neurologic deficit and achieve good seizure outcome (18). We found IONM affordable and feasible method for complex epilepsy surgeries in a setting with limited resources (Table 2). Based on our, though small, experience we believe that tailored disconnective surgeries in carefully selected patients with unilateral MRI pathology and EEG abnormalities could be a successful and safe intervention without intraoperative neuroimaging, and is essentially dependent on the neurosurgeon’s experience and skills.

### Seizure outcome

The best postsurgical outcomes in our cohort were observed in the “residual lesions” group (100% seizure freedom, three out of three children) followed by “pure” temporal lobe epilepsy (89% seizure freedom, 17 out of 19 patients). The first result is not surprising as residual hemispheric epilepsy is usually treated by large disconnective surgery, primarily hemispherotomy, which is one the most effective surgical procedure with pooled seizure-free rate of 73% in a recent meta-analysis (19). In general, the efficacy rate of epilepsy surgery in our cohort is comparable with that of other centers having the best outcomes in electro-clinically concordant MRI lesions (60–70% seizure freedom) (12, 13). In accordance with international data (13), we found that in our seizure-free group (*n* = 24) temporal lobe surgery prevailed (68%, *n* = 17) as well as shorter epilepsy duration (68%, 17 patients with surgery within 10 years after epilepsy onset).

Regarding drug discontinuation after successful epilepsy surgery, which was possible to achieve in almost 40% (*n* = 11) of our patients at 1–5 years post-operation, this result is again consistent with recent findings (14–51% drug freedom at 5 years) in the large retrospective multicenter European study (14), including almost twice as big proportion of seizure-free and drug-free pediatric cases (7 children vs. 4 adults).

All seizure-free or improved patients and/or their relatives reported marked improvement in neuropsychological functioning, which, unfortunately, cannot be objectivize by formal post-operative neuropsychological testing for previously mentioned reasons. Nearly half of our adult patients were able to secure employment.

### Complications

Complication rates for focal resective epilepsy surgery have decreased dramatically over time. Minor and major medical complications were reported in 5.1 and 1.5% of patients, respectively, and perioperative mortality was very low after epilepsy surgery, occurring in only 0.4% of temporal and 1.2% of the extratemporal epilepsy patients (20). In particular, the risk of permanent neurological deficits was very low (5.2% in temporal versus 19.5% in extratemporal group) (21) and can be explained by the use of intraoperative monitoring (18), which, we believe is the reason for lack of any persistent neurological deficit in our group.

It has been established that the frequency and nature of specific complications of epilepsy surgery vary depending on the type and extent of surgery (disconnective, resective, and invasive exploration), the location of the resection (temporal vs. extratemporal), and the age of patient (pediatric vs. adult) (20, 22). Not surprisingly, in our cohort, the most serious complications (increased intracranial pressure) occurred in two (out of 4) children with large disconnections and fortunately, only one of them (25%) needed a permanent ventriculo-peritoneal anastomosis. This incidence is similar to the serious complications rate (between 5.2 and 27.5%) in hemispherotomies found by bigger and more experienced centers (20).

The complication rate for wound infection/meningitis dropped to 1.1% in temporal and 1.9% in extratemporal surgeries as found by a large meta-analysis for the period 1980–2012 (23). In pediatric population infectious complications rate are even lower: 0.2% for osteomyelitis, 0.4% for wound infection (significantly lower than 1.1% in adults) (24), and 0.7% for meningitis (25). In our cohort infectious complications occurred in three patients (11%), which is considerably higher than the above rates (4) but remained without any sequelae. It is well known, that the rate of surgical wound infections is strongly influenced by operating theater quality (26) and appropriate post-surgical care. Surgical site infection rates can be improved by acting upon various factors—from the surgical environment itself to procedural aspects and staff behavior. We appreciate that the higher infectious complications rate in our cohort is most probably related to all these factors, as epilepsy and functional brain surgery were performed for the first time in our hospital and in the country, and environmental improvement and staff education were achieved throughout the development of our epilepsy surgery program.

### Future perspectives

Similarly to other epilepsy surgery programs, where patients and their caregivers bear the cost of medical care, majority of our patients were not able to afford expensive investigations (3, 10), this way preventing the more rapid implementation of new technologies in difficult case scenarios, which then could be considered surgically remediable and proceed to surgery. With more experience and the availability of newer technologies, more complex cases could be selected and operated. The recently acquired Electa Leksell Stereotactic System will facilitate our next step—the implementation of stereo-EEG explorations in our epilepsy surgery program.

## Conclusion

In general, the seizure outcome and general post-surgical complication rate (except the minor infectious complications) in our cohort align with international results (20). We believe that, although small, yet encompassing patients along the usual age spectrum and with the most frequent pathologies of drug-resistant epilepsies, our experience can serve as a model to develop epilepsy surgery in countries with limited resources.

## Data availability statement

The original contributions presented in the study are included in the article/supplementary material, further inquiries can be directed to the corresponding author.

## Ethics statement

The studies involving humans were approved by The Ethics Committee of Arabkir Medical Center and Institute for Child and Adolescent Health. The studies were conducted in accordance with the local legislation and institutional requirements. The ethics committee/institutional review board waived the requirement of written informed consent for participation from the participants or the participants’ legal guardians/next of kin because The Ethical Review Board determined that the study meets specific criteria for consent waiver, such as minimal risk or involvement of de-identified data. Written informed consent was obtained from the individual(s), and minor(s)’ legal guardian/next of kin, for the publication of any potentially identifiable images or data included in this article.

## Author contributions

BS and IT: study conception and design. AB, SB, AG, KM, KG, BS, and PD: data collection. PD, BS, and KM: analysis and interpretation of results. BS, KM, SB, KG, AG, IT, AB, and PD: draft manuscript preparation. All authors contributed to the article and approved the submitted version.

## Conflict of interest

The authors declare that the research was conducted in the absence of any commercial or financial relationships that could be construed as a potential conflict of interest.

## Publisher’s note

All claims expressed in this article are solely those of the authors and do not necessarily represent those of their affiliated organizations, or those of the publisher, the editors and the reviewers. Any product that may be evaluated in this article, or claim that may be made by its manufacturer, is not guaranteed or endorsed by the publisher.

## Footnotes

^1^ data.worldbank.org/

## References

[1] Forsgren L Beghi E Oun A Sillanpaa M. The epidemiology of epilepsy in Europe—a systematic review. Eur J Neurol. (2005) 12:245–53. doi: 10.1111/j.1468-1331.2004.00992.x, PMID: 15804240

[2] Fattorusso A Matricardi S Mencaroni E Dell'Isola GB Di Cara G Striano P. The Pharmacoresistant epilepsy: an overview on Existant and new emerging therapies. Front Neurol. (2021) 12:674483. doi: 10.3389/fneur.2021.674483, PMID: 34239494 PMC8258148

[3] Marashly A Karia S Zonjy B. Epilepsy surgery: special circumstances. Semin Pediatr Neurol. (2021) 39:100921. doi: 10.1016/j.spen.2021.100921, PMID: 34620459

[4] Dua T de Boer HM Prilipko LL Saxena S. Epilepsy Care in the World: results of an ILAE/IBE/WHO global campaign against epilepsy survey. Epilepsia. (2006) 47:1225–31. doi: 10.1111/j.1528-1167.2006.00595.x, PMID: 16886987

[5] Kwan P Arzimanoglou A Berg AT Brodie MJ Allen Hauser W Mathern G. Definition of drug resistant epilepsy: consensus proposal by the ad hoc task force of the ILAE commission on therapeutic strategies. Epilepsia. (2010) 51:1069–77. doi: 10.1111/j.1528-1167.2009.02397.x, PMID: 19889013

[6] Minotti L Montavont A Scholly J Tyvaert L Taussig D. Indications and limits of stereoelectroencephalography (SEEG). Neurophysiol Clin. (2018) 48:15–24. doi: 10.1016/j.neucli.2017.11.006, PMID: 29352627

[7] Engel J Cascino GD Ness PCV Rasmussen TB Ojemann LM. Outcome with respect to epileptic seizures In: Engel J, editor. Surgical Treatment of the Epilepsies. NY: Raven Press (1993)

[8] Schijns OE Hoogland G Kubben PL Koehler PJ. The start and development of epilepsy surgery in Europe: a historical review. Neurosurg Rev. (2015) 38:447–61. doi: 10.1007/s10143-015-0641-3, PMID: 26002272 PMC4469771

[9] Jukkarwala A Baheti NN Dhakoji A Salgotra B Menon G Gupta A. Establishment of low cost epilepsy surgery centers in resource poor setting. Seizure. (2019) 69:245–50. doi: 10.1016/j.seizure.2019.05.007, PMID: 31121549

[10] Radhakrishnan K. Challenges in the management of epilepsy in resource-poor countries. Nat Rev Neurol. (2009) 5:323–30. doi: 10.1038/nrneurol.2009.53, PMID: 19455183

[11] Rathore C Radhakrishnan K. Epidemiology of epilepsy surgery in India. Neurol India. (2017) 65:S52–9. doi: 10.4103/neuroindia.NI_924_16, PMID: 28281496

[12] Cohen-Gadol AA Wilhelmi BG Collignon F White JB Britton JW Cambier DM. Long-term outcome of epilepsy surgery among 399 patients with nonlesional seizure foci including mesial temporal lobe sclerosis. J Neurosurg. (2006) 104:513–24. doi: 10.3171/jns.2006.104.4.513, PMID: 16619654

[13] de Tisi J Bell GS Peacock JL McEvoy AW Harkness WF Sander JW. The long-term outcome of adult epilepsy surgery, patterns of seizure remission, and relapse: a cohort study. Lancet. (2011) 378:1388–95. doi: 10.1016/S0140-6736(11)60890-8, PMID: 22000136

[14] Lamberink HJ Otte WM Blümcke I Braun KPJ Aichholzer M Amorim I. Seizure outcome and use of antiepileptic drugs after epilepsy surgery according to histopathological diagnosis: a retrospective multicentre cohort study. Lancet Neurol. (2020) 19:748–57. doi: 10.1016/S1474-4422(20)30220-9, PMID: 32822635

[15] Giulioni M Marucci G Pelliccia V Gozzo F Barba C Didato G. Epilepsy surgery of "low grade epilepsy associated neuroepithelial tumors": a retrospective nationwide Italian study. Epilepsia. (2017) 58:1832–41. doi: 10.1111/epi.13866, PMID: 28804898

[16] Ristic AJ Mindruta I Dimova P Kelemen A Grujicic D Ilic R. Low-grade epilepsy-associated tumour management with or without presurgical evaluation: a multicentre, retrospective, observational study of postsurgical epilepsy outcome. Epileptic Disord. (2020) 22:555–62. doi: 10.1684/epd.2020.1195, PMID: 32985985

[17] Raghavendra S Nooraine J Mirsattari SM. Role of electroencephalography in presurgical evaluation of temporal lobe epilepsy. Epilepsy Res Treat. (2012) 2012:204693. doi: 10.1155/2012/204693, PMID: 23198144 PMC3503287

[18] Minkin K Gabrovski K Karazapryanov P Milenova Y Sirakov S Karakostov V. Awake epilepsy surgery in patients with focal cortical dysplasia. World Neurosurg. (2021) 151:e257–64. doi: 10.1016/j.wneu.2021.04.021, PMID: 33872840

[19] Hu WH Zhang C Zhang K Shao XQ Zhang JG. Hemispheric surgery for refractory epilepsy: a systematic review and meta-analysis with emphasis on seizure predictors and outcomes. J Neurosurg. (2016) 124:952–61. doi: 10.3171/2015.4.JNS14438, PMID: 26495944

[20] Hader WJ Tellez-Zenteno J Metcalfe A Hernandez-Ronquillo L Wiebe S Kwon CS. Complications of epilepsy surgery: a systematic review of focal surgical resections and invasive EEG monitoring. Epilepsia. (2013) 54:840–7. doi: 10.1111/epi.12161, PMID: 23551133

[21] Tebo CC Evins AI Christos PJ Kwon J Schwartz TH. Evolution of cranial epilepsy surgery complication rates: a 32-year systematic review and meta-analysis. J Neurosurg. (2014) 120:1415–27. doi: 10.3171/2014.1.JNS131694, PMID: 24559222

[22] Gooneratne IK Mannan S de Tisi J Gonzalez JC McEvoy AW Miserocchi A. Somatic complications of epilepsy surgery over 25 years at a single center. Epilepsy Res. (2017) 132:70–7. doi: 10.1016/j.eplepsyres.2017.02.016, PMID: 28324680

[23] Rugg-Gunn F Miserocchi A McEvoy A. Epilepsy surgery. Pract Neurol. (2020) 20:practneurol-2019-002192–14. doi: 10.1136/practneurol-2019-002192 31420415

[24] d'Orio P Rizzi M Mariani V Pelliccia V Lo Russo G Cardinale F. Surgery in patients with childhood-onset epilepsy: analysis of complications and predictive risk factors for a severely complicated course. J Neurol Neurosurg Psychiatry. (2019) 90:84–9. doi: 10.1136/jnnp-2018-318282, PMID: 30100551

[25] Panigrahi M Vooturi S Jayalakshmi S. Complications of epilepsy surgery: a single Surgeon's experience from South India. World Neurosurg. (2016) 91:16–22. doi: 10.1016/j.wneu.2016.03.068, PMID: 27032520

[26] Humphreys H. Preventing surgical site infection. Where now? J Hosp Infect. (2009) 73:316–22. doi: 10.1016/j.jhin.2009.03.028, PMID: 19700219

